# Probiotics and Plant-Based Foods as Preventive Agents of Urinary Tract Infection: A Narrative Review of Possible Mechanisms Related to Health

**DOI:** 10.3390/nu17060986

**Published:** 2025-03-11

**Authors:** Ariana Saraiva, Dele Raheem, Poly Rani Roy, Mona N. BinMowyna, Bernardo Romão, Sehad N. Alarifi, Najla A. Albaridi, Zayed D. Alsharari, António Raposo

**Affiliations:** 1 Research in Veterinary Medicine (I-MVET), Faculty of Veterinary Medicine, Lisbon University Centre, Lusófona University, Campo Grande 376, 1749-024 Lisboa, Portugal; ariana.saraiva@ulusofona.pt; 2 Global Change Research, Arctic Centre, University of Lapland, 96101 Rovaniemi, Finland; braheem@ulapland.fi; 3 Department of Chemistry, Jagannath University, Dhaka 1100, Bangladesh; polyrani92@gmail.com; 4 College of Education, Shaqra University, Shaqra 11911, Saudi Arabia; m.mwena@su.edu.sa; 5 Faculty of Health Sciences, Department of Nutrition, University of Brasília, Brasília 70910-900, Brazil; bernardo.lima@unb.br; 6 Department of Food and Nutrition Science, Al-Quwayiyah College of Sciences and Humanities, Shaqra University, Shaqraa 11971, Saudi Arabia; snalarifi@su.edu.sa; 7 Department of Health Science, College of Health and Rehabilitation, Princess Nourah bint Abdulrahman University, P.O. Box 84428, Riyadh 11671, Saudi Arabia; naalbaridi@pnu.edu.sa; 8 Department of Clinical Nutrition, Faculty of Applied Medical Sciences, University of Tabuk, P.O. Box 741, Tabuk 71491, Saudi Arabia; zalsharari@ut.edu.sa; 9 CBIOS (Research Center for Biosciences and Health Technologies), Universidade Lusófona de Humanidades e Tecnologias, Campo Grande 376, 1749-024 Lisboa, Portugal

**Keywords:** antimicrobial resistance, bioactive compound, fermented foods, functional foods, plant-based foods, probiotics, urinary tract infection

## Abstract

Urinary tract infections (UTIs) are a prevalent global health issue, often requiring antibiotic treatment, which contributes to antimicrobial resistance. This narrative review explores the potential of probiotics and plant-based foods as alternative or complementary preventive strategies against UTIs. Fermented foods, such as yogurt, kefir, and kombucha, contain probiotic strains that can modulate the gut and urogenital microbiota, enhancing resistance to uropathogens. Likewise, plant-based foods, including cranberry, garlic, bearberry, juniper, and nettle, possess bioactive compounds with antimicrobial, anti-inflammatory, and diuretic properties. Laboratory and clinical studies suggest that these natural interventions may reduce the incidence of UTIs by inhibiting pathogen adhesion, modulating immune responses, and promoting urinary tract health. However, despite promising findings, inconsistencies in study methodologies, dosage standardization, and long-term efficacy warrant further investigation. Future research should focus on optimizing probiotic formulations, standardizing plant-based supplement dosages, and assessing potential food–drug interactions to establish evidence-based guidelines for UTI prevention.

## 1. Introduction

A urinary tract infection (UTI) is characterized by atypical microbiological colonization of any of the component parts of the human urinary tract, such as the kidneys, ureters, bladder, and urethra [1]. This, in turn, can also be classified according to the location of the affected structure. A lower UTI is present in the bladder, while an upper UTI is present in the ureters and kidneys [1]. The pathogenesis of a UTI involves the presence of different groups of microorganisms. Around 85% of UTI cases result from the colonization of bacteria, which, in turn, commonly originate from the vaginal or intestinal canal [2]. The strains of *Escherichia coli* account for the largest percentage of infections from these sources [3,4].

There are several forms of infection in the context of this disease. In the case of lower urinary tract infections, the main entry point for bacteria in both men and women is the urethral opening, which is located on the penis and vulva, respectively [5,6].

Nevertheless, urinary tract infections are present in both men and women, and epidemiological data indicate that women are significantly more affected, due to the reduced size of their urethral canal compared to men, which favors the development and colonization of bacteria, in addition to the proximity of the entrance of the female urethral canal to the anus, which can also mainly be a source of *E. coli* contamination, because it contains traces of fecal matter [6,7].

The data show that more than 404.6 million people experience urinary tract infections worldwide, among which young women between 20 and 30 years old are the most affected, with the mortality rate estimated at 236,786 [8]. Furthermore, the populations of Latin America and tropical Asia stand out as the most affected, with the highest number of incidences [8,9]. Urinary tract infection, in this sense, constitutes a public health problem, which requires actions that are aimed at both its treatment and its prevention.

Probiotics are defined as bacteria whose presence in the human body is related to beneficial effects on health [10]. They can be administered in isolation, in capsules with stabilized and freeze-dried colonies, or in fermented foods. Multiple studies have already reported a wide range of applications of probiotics in the prevention of diseases, such as diabetes, inflammatory bowel disease, obesity, and, more recently, urinary tract infections [11,12]. The main hypothesis in regard to UTI prevention is related to an increase in the diversity of the intestinal and vaginal microbiota, which can reduce the incidence of infections through competition with pathogenic strains [13,14].

Furthermore, bioactive compounds present in plant species also have antibiotic, antioxidant, and anti-inflammatory potential within the same context, due to their ability to strengthen innate protection against pathogenic species, and thus act as a preventive measure against UTIs [15,16,17].

However, there is a gap in the knowledge regarding their effectiveness, as well as their administration doses, despite the well-known empirical applications of both classes of products. There is a lack of consensus regarding the existence of benefits that are related to the introduction of specific strains, or whether the simple intake of fermented foods can result in benefits that are similar to isolated supplementation with regard to probiotics [11].

Furthermore, there is a need to systematize knowledge about the benefits that are obtained from ingesting fresh foods, and, in the case of formulations of isolated compounds, which is the best form of presentation, dose, and frequency of treatment in regard to plant-based foods that are rich in bioactive compounds [17].

In this sense, the objective of this narrative review is to analyze the mechanisms related to possible health effects described in the literature, in the context of the use of probiotics and plant-based bioactive substances with the aim of preventing UTIs.

## 2. Probiotic Foods

Humans are known to lack the enzymes required to degrade the bulk of dietary fibers during digestion prior to excretion in the urinary tract. These nondigestible carbohydrates pass through the upper gastrointestinal tract unaffected, and they are fermented in the cecum and the large intestine by the anaerobic cecal and colonic microbiota [18]. Probiotics produce antimicrobial substances, such as bacteriocins and short-chain fatty acids (SCFAs), which may inhibit the growth of potential pathogens [18].

The mechanism of action that confers immunity to microbial infection can be complex. Toll-like receptors (TLRs) are regarded as an important family of receptors that constitute the first line of defense against microbes. TLRs are molecules that alert the immune system when there are microbial infections [19].

The existence of the common mucosal immune system makes oral probiotic supplementation an effective method to influence the mucosal sites in a different way to intestinal microorganisms. After intestinal antigens are delivered to Peyer’s patches, both B and T cells will migrate from the Peyer’s patches to the mucosal membranes of the respiratory, gastrointestinal, and genitourinary tracts, as well as to exocrine glands, such as the lacrimal, salivary, mammary, and prostatic glands [20].

Previous studies have shown that Lactobacillus species, in the form of probiotics, reduce the incidence of UTIs and vaginal infections [21]. However, the important mechanisms for the partial amelioration of UTIs in various models seem to involve downregulating the production of proinflammatory cytokines (interleukin-6 [IL-6], IL-8, and the tumor necrosis factor alpha) [22]. Clinical evidence of antimicrobial activities by isolated probiotics, and their modes of action, is shown in Table 1.

Another clinical study also reported that fecal microbiota transplantation by a donor stool transplant, which involves the action of bacterial interference, led to a significant reduction in rUTIs [29].

However, besides the use of isolated probiotic strains, possible health benefits may be gained by consuming food and drinks which have undergone fermentation with beneficial strains, as shown in Table 2 below [21].

It is possible that multi-strain or multi-species probiotic formulations have greater efficacy in fighting infections when compared to single-strain administration, as a result of the complementary or synergistic effects of multi-strain/species formulations [33]. Even though a good number of LAB originating from fermented dairy foods and the intestinal tract of humans or animals have been widely characterized as having an antagonistic ability with probiotic potential, there has been less attention directed to LAB that are derived from plant-based fermented foods [34]. Some of these antagonistic abilities include adhesion to the intestine, reduction of pathogenic bacterial adhesion to the intestine, aggregation, and coaggregation, as well as the production of antimicrobial substances such as bacteriocins [35].

These bacteria and yeasts are regularly employed, either as single strains or mixed strains, in order to produce some well-known probiotic foods and drinks, which are described below with their possible mechanisms related to health and prevention of infections.

### 2.1. Kefir

Kefir is a fermented probiotic drink made by adding kefir grains to cow’s or goat’s milk, or even fruit beverages. Characterized as cultures of lactic acid bacteria and yeast, kefir grains are composed of a complex polysaccharide and protein matrix [36].

The bioactive compounds that are present in kefir have been shown to present antimicrobial, anticancer, and immune-modulatory activities [37]. In addition, the exopolysaccharides produced by kefir possess antioxidant properties [38]. Therefore, they are able to provide complete resistance against hydrogen peroxide by reversing its detrimental effect on the cell growth of the microorganisms present in kefir [38].

In this sense, the effects caused by kefir consumption on the composition of the intestinal microbiota may be due to a combination of factors, such as direct pathogen inhibition by acids, and bacteriocin production, in addition to competitive pathogen exclusion in the intestinal mucosa [39].

According to Marquina et al., kefir consumption significantly increases lactic acid bacteria counts in the intestinal mucosa, and reduces enterobacteria and clostridia populations [40]. Furthermore, kefir consumption was also demonstrated to prevent *C. jejuni* colonization in chick ceca [39], and it has been shown to be effective in postoperative treatments and in patients with gastrointestinal disorders [41].

### 2.2. Kombucha

Kombucha is a fermented black or green tea drink that is popular in many parts of the world, especially in Asia. Kombucha is fermented by creating an infusion of sweetened black tea, followed by incorporating a starter culture, known as the Symbiotic Culture of Bacteria and Yeast (SCOBY), for a period of 7 to 10 days [42].

Sucrose from the medium is first hydrolyzed to glucose and fructose simple sugars by (β-fructofuranosidase, EC 3.2.1.26), which is an invertase enzyme that is primarily produced by yeast species, such as S. cerevisiae.

Yeasts synthesize ethanol and carbon dioxide as metabolites from the resultant monosaccharides, which are then oxidized by acetic acid bacteria in order to produce acetic acid over the following days [43]. Probiotics isolated from kombucha and kefir are bacteria, which include *Lb. acidophilus*, *Lc. casei*, *Lc. rhamnosus*, *Bifidobacterium lactis*, and *Bacillus coagulans*, and yeasts, which include *Km. marxianus*, *S. cerevisiae*, and *S. boulardii*. Metabolites that are produced by microbes during fermentation, which include proteolytic enzymes, organic acids such as glucuronic acid, and exopolysaccharides, further enhance health [43].

These metabolites and their antioxidant properties can enhance health by preventing cardiovascular diseases (CVDs) through their actions of low-density lipoprotein (LDL) oxidation, regulation of cholesterol metabolism, and aid in smooth muscle relaxation, which eventually lead to lowering blood pressure [44,45].

### 2.3. Yogurt

Yogurts are made from milk that is fermented by probiotics, which mainly include lactic acid bacteria and bifidobacteria. The immune-promoting activity is most likely derived from the additive or synergistic actions of bioactive components, which makes it difficult to disentangle the specific contribution of actives. Furthermore, the number of fermentation-derived bioactive peptides and amino acids increases during storage, mainly due the action of microorganisms [46].

The specific actions of some of these bioactives are known. For example, yogurt contains antimicrobial substances, such as bacteriocins, which are effective against pathogenic microorganisms, and they may help in preventing infections [47].

### 2.4. Miso

Miso is a fermented soybean paste and a popular Japanese seasoning, which is formed by combining two fermentation processes.

Firstly, koji is a substrate that is first inoculated with a mold, usually *Aspergillus oryzae*, and then fermented. Secondly, koji is mixed with salt and soybean mash, and fermented again by yeast and bacteria, which results in miso [48].

The microbial community of koji and miso is thought to be crucial to the formation of miso’s unique flavor, texture, and nutritional composition during fermentation. Miso’s isoflavones and phenolic acids, which include 8-OH-daidzein, 8-OH-genistein, 6-OH-daidzein, and syringic acid, have stronger antioxidant activity than α-tocopherol as a probiotic food [49]. 

### 2.5. Sauerkraut

Sauerkraut is produced by the fermentation of finely shredded cabbage with lactic acid bacteria. It is a traditional food that is popular in many countries, especially in Eastern Europe [50].

Sauerkraut is made through spontaneous fermentation by yeast and fungus, in addition to lactic acid bacteria, which results in microbiological, metabolic, and physiological changes that alter the integrity and quality of the final product, which often has a sour and salty taste [50].

Sauerkraut possess a number of acclaimed health benefits, due to its concentrations of vitamin C, vitamin B, and minerals, which include iron, calcium, potassium, phosphorus, and phenolic compounds [51].

The health-promoting properties of sauerkraut, such as its ability to exert anti-inflammatory, antioxidant, and anticarcinogenic activities that protect against oxidative DNA damage, are strongly supported by scientific research [52]. Also, some of the glucosinolate (a sulfur glycoside molecule found in *Brassica* vegetables) hydrolysis products found in sauerkraut, including allyl isothiocyanate and phenyl isothiocyanate, have been shown to exhibit antioxidant properties in vitro [53].

Owing to the high concentration of vitamin C and E and other phenolic compounds found in sauerkraut, they collectively serve as powerful free radical scavengers, protecting the body against oxidative stress. Vitamin C and phenolic compounds are both antioxidants that help to protect against the effects of free radicals [53,54]. Vitamin C lowers inflammation and atherosclerotic plaque disruption, due to the C-reactive protein, and acts as an electron donor for eight human enzymes, neutralizing superoxide and hydroxyl radicals [55]. Due to its ability to donate a hydrogen atom, vitamin E exhibits antioxidant activity, protecting against cardiovascular disease by inhibiting low-density lipoprotein oxidation [56].

### 2.6. Tempeh

Tempeh is a fermented soybean product that forms a firm patty. It originates from Indonesia, and has become popular worldwide as a high-protein meat substitute.

It is often described as having a nutty and earthy flavor, similar to the flavor of mushrooms. Tempeh is usually made by fermenting soybeans with *Rhizopus* spp., but it can be made using various nuts, grains, and beans [57].

It is well known as a source of significant amounts of protein, Vitamin B12, and bioactive compounds [58]. The evaluation of acidification and lactic acid bacteria co-inoculation in the soaking process is noted to be a critical way to further improve the safety of tempeh production in the industry [59].

### 2.7. Natto

Natto is a staple in Japanese kitchens. Usually, it is consumed with rice as a common Japanese breakfast. Natto is made from soybeans that are cooked and inoculated with *Bacillus subtilis* for fermentation.

It has a distinctive smell, slippery texture, and strong flavor. Regarding its possible health benefits, natto presents many bioactive components generated during the fermentation process, such as nattokinase (NK), bacillopeptidase F (BPF), vitamin K2 (menaquinone-7), dipicolinic acid (DPA), and γ-polyglutamic acid (γ-PGA), which have the potential to increase immunity to infections [60]. 

### 2.8. Kimchi

Kimchi is a fermented and spicy Korean side dish. The vegetables that are the most frequently used to make kimchi are baechu cabbages (*Brassica rapa*) and radishes (*Raphanus raphanistrum*). Other vegetables, such as cucumbers, spring onions, and other plants, are also widely used, which results in hundreds of different types of kimchi being consumed in Korea [61,62].

The fermentation of kimchi involves numerous microorganisms, such as lactic acid bacteria (LAB), and the microbial composition of kimchi differs based on the type and amount of the ingredients. LAB that are commonly used in kimchi include species of the genera *Lactobacillus*, *Leuconostoc*, and *Weissella* [61,62].

In general, fermented foods contain substances associated with health; however, it is important to note that confirming the role of probiotic foods as a preventive measure against urinary tract infections will require additional clinical trials, in order to evaluate the safety and efficacy of probiotics in different populations, and to identify their potential interactions with other medications. Studies are needed to explore the use of probiotics in combination with antibiotics, as well as to evaluate the long-term effects of probiotic use.

As for probiotics in their isolated presentations, there are still challenges regarding the availability of access to high-quality probiotic products, the selection of appropriate strains, and the lack of consensus regarding optimal dosing and the duration of probiotic use [63].

Future research should focus on identifying the optimal probiotic strains and regimens for the prevention and treatment of UTIs, gaining a better understanding of the role of the gut microbiota in regard to urogenital health, and developing new probiotic technologies and delivery methods. A recent review suggests that there is a limited body of evidence that supports the implementation of probiotics as part of the management strategy for recurrent UTIs [13]. The authors advocate the need for further trials in a multicenter setting, which include larger sample sizes and the implementation of standardized parameters.

## 3. Plant-Based Foods and Supplementation

### 3.1. Cranberry

*Vaccinium macrocarpon*, *Vaccinium oxycoccos*, and *Vaccinium erythrocarpum* are the scientific names of cranberry [64,65]. Multiple health benefits are associated with the consumption of cranberries to prevent UTIs. The consumption of fresh cranberries or cranberry juice has been indicated to prevent the risk of UTI manifestation in women who have recurrent urinary tract infections (UTIs) [66,67], and in healthy women, as a natural alternative [68,69].

Moreover, the European Association of Urology recommends the consumption of cranberries as a way to successfully avoid UTIs [70].

In fact, in the research of Xia et al. [70], women who frequently experienced UTIs, children, and individuals using indwelling catheters had a relative risk reduction of 32%, 45%, and 51%, respectively. Better results were shown (reduction of 35% in relative risk) when cranberry juice was consumed instead of cranberry tablets being ingested. In this sense, cranberry might be considered an auxiliary therapy for preventing UTIs in susceptible populations [59].

Since the 1900s, the mechanism behind the positive effect of cranberry consumption on UTIs had been attributed to a decrease in urine pH, which is due to cranberry’s content of organic acids and phenolic compounds [71,72,73]. *Enterobacteriaceae*, which is one of the pathogens that is responsible for promoting UTIs, is more likely to colonize when the pH of urine is increased [74]. Compounds that are present in cranberry juice, such as malic, quinic, and shikmic acid, promote a low pH of 2.5, and thus produce an unfavorable environment for the development of Gram-negative bacteria [75,76]. Hippuric acid, which possesses potent antibacterial qualities, is also produced through the metabolism of quinic acid [77].

A study performed in 2017 using mice showed that a group that consumed cranberry juice had a lower urinary pH (5.8) compared with a group of mice that drank water (6.5). The study concluded that cranberry juice acidified the urine [77]. This observation was supported by another study conducted on a small sample of 12 healthy male subjects, where, once again, the consumption of cranberry juice (330 mL/day) acidified the urine. The pH of urine was 6.35 before consumption, and it was 6.18 after consumption. According to this study, no more than 550 mL of cranberry juice should be consumed per day, due to the high amount of calories in cranberry juice (350 kcal/l) [78].

This was the first attempt to explain the mechanism whereby cranberry appears to demonstrate positive effects against UTIs; however, researchers began to have doubts about the link between the acidification of urine and the bacteriostatic environment. In addition, consumers have to ingest large amounts of cranberry in order to observe a decrease in their urine pH [72,73].

Other important characteristics of cranberries that began to be focused on were their high number of important compounds, including polyphenols [6,72,79,80,81,82,83,84] such as proanthocyanidins (PACs). This knowledge led to a new proposed mechanism of action, with researchers starting to link PACs with their protective effects against UTIs [6,79,80,81]. In fact, several studies reported that cranberries have polyphenols that block the union of UTI bacteria with uroepithelial cell receptors [6,72,79,80,81,82,83,84].

This may be the case for infections involving *Escherichia coli*, one of the main bacteria responsible for UTIs [85,86], given that this bacterium’s adhesins, such as fimbriae adhesins like P fimbriae and pili type I, link with the surface of host cells and enable the bacterium to remain permanently in the urinary tract [9,80].

A study performed in vitro showed that cranberry phenols prevent the colonization of *E. coli* in the gut, which prevents UTIs [87]. *E. coli* can easily migrate from the gut to the urinary tract when the intestinal impermeability suffers disruption [83]. However, the capability of proanthocyanidins to link to the surface of virulence factors, such as P fimbriae from *E. coli*, that are responsible for host colonization blocks their adhesion to the urinary tract. This occurs because proanthocyanidins are analogs of the urinary tract receptors [64,87,88]. *E. coli* bacteria can only promote uropathogens if they bind with mannosylated proteins (uroplakins) [89,90]. Bacteria will not develop or cause adverse effects if they cannot bind to these cells [91,92,93,94,95].

According to Hisano et al. and Ghosh et al., most strains of *E. coli* exhibited a 75% reduction in adherence to epithelial cells when exposed to 50 μg/mL of cranberry extract [76,96].

Güven et al. reported that consumption of a cranberry extract (514 mg) which had 36 mg of PACs produced the same effect as that seen in another group that took fosfomycin [74]. The amount of leukocytes in the urine was, in fact, almost the same in the two groups. This may enable UTIs to be cured without the use of medicines [74].

Moreover, older studies correlating the consumption of cranberry juice and UTIs have already shown that this fruit can be a good ally in regard to preserving the health of the urinary tract [97,98]. For example, a study conducted in 2022 showed that 10 h after the ingestion of 240 mL of cranberry juice, the adhesion of *E. coli* was inhibited by about 80% [98,99].

A recent study in vitro with cranberry extract also confirmed that through the action of its pholyphenols, it can help to improve the function of the gut and urinary tract barriers, preventing *E. coli* infections. However, more studies are necessary in order to fully comprehend the mechanism of these polyphenols [81].

In addition, cranberries have fructose, which inhibits the adhesins of *E. coli* that are sensitive to mannose [98]. Fructose has an affinity to FimH, which allows the factor of virulence type I pili of *E. coli* to connect to the urinary cells of the host [100,101,102]. There are unfortunately no studies that directly link fructose to UTIs, and there are no studies yet that link cranberry’s fructose to the beneficial effects that this fruit has on UTIs.

Another important compound of cranberries is D-mannose. This is a basic sugar that is present in several fruits, and has been linked to methods of preventing UTIs since the 1970s [100,103,104,105]. Studies involving this monosaccharide have suggested that it can bind with *E. coli* bacteria and promote their elimination through the urine, which is called competitive inhibition [106]. Free D-mannose saturates the *E. coli* FimH structure, which blocks its linkage with urinary cells [89,90,100]. *E. coli* bacteria remain in the urinary tract due to their connection to type I pili via their adhesion component FimH, which is a virulence factor, through a mannose-sensitive mechanism [89,90,100]. It is important to conduct studies relating to D-mannose in cranberries, in order to assess whether its content is, in fact, relevant in regard to protecting against urinary infections.

The amount of cranberry juice required to be ingested in order to achieve effective results may be excessively high [107]; therefore, we found it necessary to look for studies that were conducted with the ingestion of supplements. In one study, encapsulated juice concentrate, which was equivalent to 8 ounces of cranberry juice, was supplemented 2× per day in patients with recurrent episodes of UTI. The main result was a reduction in UTI episodes in the group that was treated with concentrated juice capsules (OR: 0.39; CI 0.19–0.79; *p* = 0.0008) [107].

Daily supplementation with 500 mg of encapsulated dry Vaccinium marcocarpon extract reduced the incidence of UTIs in patients with neurogenic bladder, including a reduction from 10 annual episodes to just 3 [108]. Furthermore, one of the findings presented in this study was that better results were obtained in patients with higher glomerular filtration rates, which highlights the role of adequate diuresis in preventing UTIs [108].

In addition, a study on supplementation once a day with a cranberry extract standardized to have a 1.8% protoanthocyanidin content (9 mg) demonstrated a decrease in the incidence of UTIs in the elderly, from 84.8 to 62.8 per 100 people/year [21]. The study also pointed out that this type of supplementation only showed significant results in elderly people with compromised diuresis or renal function, whereas no results were found in people with preserved urinary function [21].

In conclusion, recent studies show some evidence that cranberries can be used to help prevent or treat UTIs, but more trials are needed in order to confirm the effectiveness of this fruit as an alternative to traditional antibiotics, due to the lack of solid evidence supporting these claims, such as regarding the dosage, standardization, and periodization of the treatment. There is also very little conclusive information on the adverse effects of using cranberries as a treatment.

### 3.2. Garlic

*Allium sativum* is the scientific name of garlic. This vegetable is well known for its antibiotic properties [109,110,111]. Some studies note that it may be useful in the treatment of several diseases, such as female urinary tract infections [110,112,113] caused by *Candida albicans*, which is one of the most common agents in these infections [110,114,115,116].

The therapeutic bioactives in garlic are allicin [111,117] and ajoene [117,118], which can eliminate and inhibit the growth of microorganisms [119,120]. Allicin’s release occurs when garlic is crushed, especially when it is raw [120]. This compound is formed following the reaction of alliin and the enzyme alliinase when garlic is chewed or chopped [121]. The decomposition of allicin after oxidation produces ajoene, among other compounds. [122].

Allicin prevents microorganisms from using some functions, such as RNA and lipid biosynthesis. These properties are hugely important, because all the enzymes that synthesize acetyl-CoA from acetate malfunction in bacteria as a result of their non-covalent binding [111].

According to an in vitro study, allicin showed results through its mechanism of regulating the secretion of proinflammatory factors, which is a consequence of the adhesion of strains that cause UTIs. In this case, allicin inhibited NF-κB and the interleukins IL-6 and IL-1β [111,123].

Researchers have studied the medicinal properties of garlic extract since 1977 [124]. For example, antifungal action was observed in urine and blood sera one hour after garlic extract (25 mL) consumption in a study from New Jersey, USA [125]. It was concluded that garlic can be used as a therapeutic strategy to treat these types of fungal infections. Another assay by Strika et al., with the same conclusions, was conducted in the last decade in Bosnia and Herzegovina, but this time, the experiments were performed in vitro with fresh local Kakanj garlic [116].

These authors also confirmed the sensibility of *Candida albicans* to garlic using the disk diffusion method. The researchers believed that, considering their results, *Allium sattivum* could be used in clinical practice in order to treat diseases caused by *Candida albicans*.

Oloche et al. also recently confirmed that aqueous garlic extract exhibited antifungal action against *Candida albicans* in concentrations varying from 100 mg/mL to 500 mg/mL [126]. The higher the concentration, the greater the antifungal activity. These results agree with another study by Lemar et al., who concluded that garlic can be used as a natural antifungal, and may be helpful in the treatment of urinary infections caused by *Candida albicans* [127].

The use of garlic extract, instead of consumption of the fresh plant, was investigated in most of the studies found in the literature [124,125,126,127], so we considered that it would also be relevant to also analyze studies conducted on the ingestion of fresh garlic with a focus on urinary tract infections.

Natural garlic juice was used in order to test the antifungal properties of garlic when exposed to *Candida albicans* in vitro in a study by Balach et al. [128]. They observed the inhibition of this fungi even with a 1:128 dilution, which confirms the antifungal action of garlic. However, some studies also report *E. coli* as one of the principal causes of urinary tract infections [85,86,129]. According to a study conducted by Salman, it is possible to prevent the growth of *E. coli* with the consumption of garlic infusion (100 ppt) [130]. If the concentration of the garlic infusion consumed is lower, the results decrease accordingly. These results are in accordance even with those of studies involving garlic extract [109,131,132], and as observed by Magryś et al., fresh garlic extract can also inhibit the growth of *E. coli* [131]. These researchers observed that fresh garlic extract can suppress the growth of *E. coli* at a concentration of 375 mg/mL. Inhibition of *E. coli* with concentrations between 100 mg/mL and 400 mg/mL was also observed in vitro in an assay by Garba et al., in which a methanolic extract of garlic was used [109]. These studies support the claims regarding the medicinal effects of this plant. All the studies correlate garlic with its antifungal action, but more studies are needed in order to confirm its recommended dosage.

In a study where patients were supplemented with 400 mg tablets of garlic extract, it was shown that patients in the intervention group presented no catheter infection, and their mean body temperature, which is commonly associated as an indicator of infection, was lower than that of patients in the control group. 

Although statistical significance was not considered, the cases of urinary tract infections in the group that took garlic supplements were lower than in the placebo group. This type of supplementation may be used to prevent urinary tract infections; however, a larger sample is needed in order to solidify these results [133].

The consumption of garlic is safe for humans, but consumers may experience an upset stomach when ingesting high amounts of it, according to the US Food and Drug Administration (FDA), which assesses food safety. 

Randomized controlled assays have concluded some side effects of garlic consumption. The secondary symptoms that have been observed include insomnia, dizziness, emesis, tachycardia, headaches, flushing, mild orthostatic hypotension, sweating, discomfort, foul body odor, and defecations [134,135]. Fresh garlic can provoke changes in the intestinal flora, flatulence, and bloating when large amounts are ingested not as part of a meal [135]. Nevertheless, the amount of garlic consumption required in order to induce a positive effect can differ according to health problems and age. Further investigations are needed in order to define the amount that should be ingested.

### 3.3. Bearberry

*Arctostaphylos uva-ursi* is commonly known as bearberry (Yarnell, 2002) or uplant cranberry [79]. The consumption of bearberry has been associated with the treatment of UTIs [79,136,137] due to its diuretic functions [137,138,139]. Bearberry contains arbutin and antioxidants, such as ferulic acid, catechin [140], gallic acid, caffeic acid, and ellagic acid, among other compounds, that can be crucial in preventing UTIs [79,141]. Bearberry has been approved in Germany as a good ally against urinary infections that are provoked by *E. coli* [142].

One of the mechanisms that has been proposed to explain the preventive action of bearberry against UTIs is the existence of glycoside arbutoside (arbutin) on its leaves, which one of its major constituents [138,139,143,144].

Glycoside arbutoside gives rise to hydroquinone glucuronide after the modification of arbutin in the intestine and liver. This compound decomposes to produce hydroquinone, which alkalizes the urine. Hydroquinone has the power to inhibit or destroy bacteria [136,138,139,143]. Consuming *A. uva-ursi* extract makes it more difficult for bacteria to form attachments in urinary systems, because it changes their surface, making them more lipidic [138]. This fruit also has gallic and ellagic acids [141,145] that promote deficiencies in the membranes of pathogens, causing their inhibition [141].

Another component of bearberry, ferulic acid, has a beneficial effect against *E. faecalis*, as it destroys the molecular structure of its cell membrane [140]. Also, catechin is reported to be a crucial element of bearberry extract, due to its function of decreasing the activity of Gram-positive bacteria and having some interference with Gram-negative bacteria [140].

One of the first studies on this plant was conducted in 1993 with 57 women. Twenty-seven women received a placebo, and the rest consumed what the authors called UVA-E (a mix of *A. uva-ursi* hydroalcoholic extract, phenolglycosides arbutin, phenolglycosides metylarbutin, and a hydroalcoholic extract of *Taraxacum officinale*), for one year in this double-blind study. The placebo group presented five cases of urinary tract infections, whereas there was no recurrence in the group that was administered UVA-E. UVA-E, which includes *A. uva-ursi*, can be considered for prophylaxis against UTIs. Due to the limitations of the study, additional research is required to determine that *A. uva-ursi* is the main cause of this beneficial effect [146].

The antibacterial activity of *A. uva-ursi* against bacteria such as *E. coli* and *S. aureus* was tested through the well diffusion method, in a study involving three groups. One group was treated with a standard drug such as ampicillin, the second with an *A. uva-ursi* mother tincture, and the other with potentized *A. uva-ursi* (30C). The results were clear, showing that, in decreasing order, the treatment with the greatest antibacterial effect was the antibiotic, followed by the *A. uva-ursi* mother tincture and the potentized *A. uva-ursi*. The antibacterial effect against *S. aureus* was strongest in all groups. In conclusion, this plant can be useful in regard to preventing urinary tract infections caused by these bacteria [147].

In a 14-day trial study conducted on rats, a 5% aqueous extract of bearberry increased the excretion of both ions from the second day of administration. The authors observed an increase in diuresis, which thus confirmed the diuretic effect of bearberry [148].

It is rare to find a studies involving the fresh fruit. However, Hafizović et al. studied tea made from the dried leaves of *A. uva-ursi*. Its antimicrobial effect was, in fact, low. According to their research, this antimicrobial effect could have been higher if they had used fresh leaves instead of dried leaves [149]. We could find no studies on *A. uva-ursi* supplementation in relation to urinary infections. 

It is important to consider the safety of bearberry consumption. Prasad advises that the prolonged ingestion of bearberry can lead to intoxication and liver or kidney problems, but the maximum safe quantities are, unfortunately, not referenced. It will be important to establish these limits in the future [150].

In vitro studies show that prolonged exposure to hydroquinone is carcinogenic; therefore, due to the hydroquinone compound that exists in bearberry, it cannot be ingested for longer than two weeks [80]. More studies are necessary to investigate the quantity that must be ingested in order to prevent or treat UTIs, as well as to confirm the maximum amount that can be safely ingested during treatment.

### 3.4. Juniper

*Juniperus communis* is the scientific name of juniper. This plant has been used since ancient times by Native Americans in order to treat UTIs [151], due to its diuretic activity [152,153,154]. The most important phytochemicals are volatile oils. The essential oil of juniper contains terpenoids (Terpinen4-ol), which is present in the leaves and berries. This compound is beneficial for the treatment of UTIs [139,155,156]. The amount of essential oil in a single berry is between 0.5 and 2.5% (V/m) [155].

Most existing laboratory studies on juniper berries are from the 1990s [154,156]. Researchers tested the essential oil from juniper berries in 1998. It was proven that juniper berries have diuretic effects in mice, due to their terpenoids [154]. Years later, researchers studied the antimicrobial effects of terpenoids on Gram-positive and Gram-negative bacteria in vitro. This led to the finding that terpenoids are effective against *Klebsiella*, *Proteus,* and *Enterococcus*, which are responsible for UTIs, but not against *E. coli* [155].

Researchers recently conducted a survey in Poland with healers and informants (N = 23), with the goal of investigating the main plants used to treat UTIs. Among a list of 123 species, juniper was included. This type of treatment is used by people who do not want to take antibiotics [152].

In conclusion, it is important to have up-to-date research on the other constituents of juniper that can explain its beneficial effect on UTIs. It is also necessary to determine the quantity that should be consumed to obtain positive effects, as well as the maximum quantity that can be ingested in order to avoid potential adverse effects on human health.

### 3.5. Urtica

*Urtica dioica* L. is commonly known as nettle. It contains a diverse range of compounds, such as flavonoids, phenolic elements, tannins, and essential oils [157,158]. This plant, which has diuretic properties, has been used to treat the symptoms of UTIs [16,157,158]. The European Commission has approved the use of this plant to decrease inflammation in the urinary tract. More specifically, the use of the flowering aerial parts and the leaves is advised [159].

Morocco is among the nations that typically utilize the entirety of this plant’s components as a diuretic [160]. The diuretic properties of nettle are given by caffeine, malic acid, and chlorogenic [160,161], which aid the excretion of toxins. Furthermore, it has been found that nettle extract has properties that make it a good ally against some Gram-positive and Gram-negative bacteria [157,162], such as *P. aeruginosa* [162], *E. coli* [157,162], and *S*. *aureus* [157,162,163]. Külcü et al. also reported that ethanol and chloroform extracted from nettle leaves could inhibit *Proteus vulgaris* and *E. faecalis* microorganisms, which are responsible for UTIs [164]. However, Mzid et al. saw no effects of ethanol extracted from nettle against *E. coli* and *E. faecalis* [165]. Most of the studies we found that directly link this plant to UTIs are not recent.

According to Wegener, in an observational study, the amount of urine excretion rose after participants consumed fresh *U. dioica* juice for 12 weeks. One hundred and fourteen patients participated in this study, and tolerance to the prolonged consumption of *U. dioica* was proven [166].

In conclusion, not many studies were found that link nettle consumption to the improvement of urinary tract infection symptoms. In addition, no studies exist on the safe consumption of nettle supplementation. However, it is also very important to ensure safety in the use of nettle consumption to promote urinary health. Some researchers, after analyzing nettle extract, have found that it does not have cytotoxic activity [167,168], which means that it will be important to conduct more research in order to guarantee the health of consumers. No intake limit has been suggested so far. Only a study on rats determined an LD50, which is the dose that is required to kill half of the test population, of 1310 mg/kg [167,168].

Due to the contradictory information that is observed in different studies, it is relevant to understand their weaknesses in order to improve methods, reduce limitations, and reach more reliable conclusions. It is also relevant to understand the impact of the consumption of this fruit in people with UTIs, in order to determine the quantities required to produce beneficial effects, as well as amount that can be safely ingested.

Table 3 highlights studies regarding the impact of plant-based foods on UTIs, including their mode of action and the phytocompounds involved.

However, it is important to highlight the lack of in vivo studies that include adequate designs, randomization, and the blinding of evaluated groups, despite promising results. Therefore, the evidence for the use of garlic in preventing UTIs is considered insufficient for its use in routine clinical applications.

Herbal therapies are widely used as cost-effective alternatives to traditional diuretics. All plants that have this effect generally act to stimulate the secretion of electrolytes at the kidney level, which thus attract water molecules and increase diuresis [17].

Increasing diuresis is one of the best-known strategies in the treatment of UTIs, which is based on the principle that more frequent urination, with increased urine flow, makes it more difficult for bacteria that cause UTIs to adhere to the uroepithelium [171].

## 4. Drug–Food Interactions

**Figure 1:**
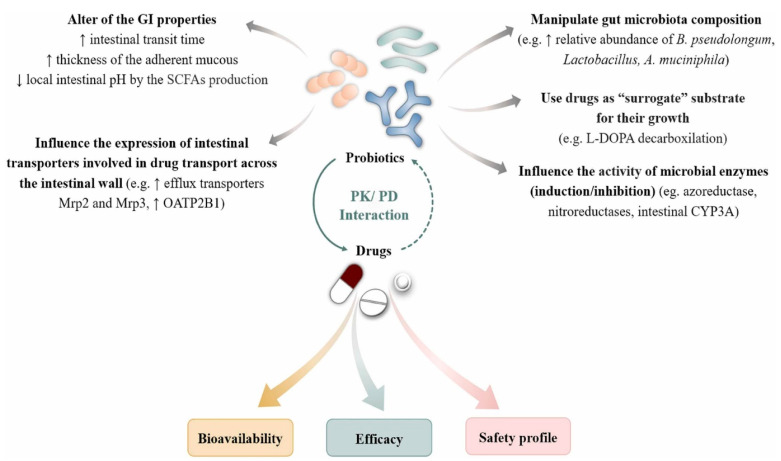
The possible mechanisms of probiotics–drug interaction. Abbreviations: GI is gastrointestinal, Mrp2 is multidrug resistance-associated protein 2, Mrp3 is multidrug resistance-associated protein 3, OATP2B1 is organic anion-transporting polypeptide 2B1, PD is pharmacodynamic, and PK is pharmacokinetic, ↑, increase ↓, decrease [172].

Probiotics have an influence on drug pharmacokinetics by affecting their absorption and composition within the gut microbiota, which can lead to an alteration in the bioavailability of drugs. Probiotics are generally regarded as safe (GRAS). However, their exact mechanisms are only partially known, so more research regarding probiotic–drug and probiotic–gut microbiota interactions is expected in the near future, due to their ever-increasing consumption, their potential influence on metabolism, and the efficacy and safety of orally administered drugs [172]. It is worth considering the influence of probiotics on the bioavailability of drugs by several possible mechanisms, as shown in Figure 1 below, in the treatment of urinary infections. The authors of [172] observed that probiotics affect the bioavailability of drugs by altering the local intestinal pH via the production of short-chain fatty acids, which increases the intestinal transit time or adherent mucous thickness, influences the expression of intestinal transporters that are involved in drug transport across the intestinal wall, manipulates the gut microbiota composition, and modulates the activity of microbial enzymes, via induction or inhibition, that use drugs as substrates for their own growth.

The role of the gut microbiota in the antitumor effects of dietary intervention was highlighted in a recent study. The enrichment of Bifidobacterium bifidum after caloric restriction specifically increased acetate levels, which, in turn, elevated the number of IFNγ+CD8+ T cells in the tumor microenvironment. The antitumor effect of IF was, in contrast, not mediated by the gut microbiome, because it was not abrogated after the microbiota was depleted [173].

Recent studies have also revealed that a ketogenic diet significantly influences the gut microbiota, inducing a shift from a population that is dominated by tolerogenic bacteria (*Lactobacilli* spp., *Clostridium asparagiforme*) toward a population that is dominated by immunogenic bacteria, such as *Akkermansia muciniphila* [174]. It has been reported that a shift in the gut microbiota in response to intake of a ketogenic diet is partially attributable to the host’s production of ketone bodies. β-HB selectively suppresses the proliferation of Bifidobacterium among these ketone bodies. This suppression subsequently leads to a reduction in intestinal Th17 immune cells [175].

Dietary methionine/cystine restriction has been shown to alter the gut microbiota, and it can contribute to immune system alterations. This type of diet restriction specifically promotes a significant decrease in the relative abundance of multiple *Ruminococcaceae* and *Prevotellaceae* families, as well as a decrease in the presence of members of the *Lactobacillaceae* family [176].

A diet rich in salt promotes an increase in the abundance of *Bifidobacterium*, which infiltrates tumors, subsequently augments the functionality of natural killer cells, and ultimately contributes to tumor regression, due to enhanced gut permeability. These results suggest that intake of a high-salt diet modulates the gut microbiome, and it may stimulate natural killer cell-dependent tumor immunity, thereby having potential implications for the development of novel therapeutic interventions [177].

A study was conducted in order to examine the association between a vegetarian diet and UTI risk in a Taiwanese Buddhist population, due to the fact that vegetarian diets comprise abundant phytochemicals, which may contain antimicrobial properties and protect against UTIs [178]. Studies have already shown that medicinal plants have broad-spectrum antimicrobial activity against uropathogenic *E. coli* and other UTI pathogens [179,180,181]. However, the strains of *E. coli* that cause UTIs, which are otherwise known as extra-intestinal pathogenic *E. coli* (ExPEC), are distinct from the intestinal pathogenic strains and normal commensal strains [182]. It is worth noting that ExPECs isolated from animal food products and UTI patients have been found to be very similar in terms of their antibiotic resistant patterns and virulence factor profiles [183,184].

The antibiotic treatment of UTIs is quite challenging, due to high recurrence rates and increasing multidrug resistance in ExPECs [185]; therefore, an alternative non-antibiotic method of UTI management is important. A healthy vegetarian diet with a high phytochemical content may constitute an alternative prophylaxis, providing a bactericidal effect against UTIs [186,187,188]. However, there is need for further studies in order to better understand the exact intake of specific phytochemicals in vegetarian diets that are known to contribute to UTI protection.

The use of antibiotics in animal agriculture may contribute to increasing antibiotic resistance in humans. Extended spectrum β-lactamase (ESBL)-producing and fluoroquinolone-resistant ExPECs, which are mostly disseminated by the *E. coli* strain O25:H4-ST131 and account for 78% of cases of antibiotic resistance globally, are a major problem, with resistance to different types of penicillin, cephalosporins, and fluoroquinolones [185,189]. In addition, the ESBLs encoded on plasmids can carry other antibiotic resistance genes against aminoglycosides, sulfonamides, and quinolones, which lead to multidrug resistance [185,189].

Drug–food interactions could result in either the induction or inhibition of enzymes in the gut by nutrients, which can lead to significant changes in oral bioavailability of drugs, or vice versa. For example, grapefruit juice is a selective intestinal CYP3A4 inhibitor, and the overall exposure of some drugs with the risk of adverse effects can be increased by more than fivefold when taken with grapefruit juice [190].

There is evidence that shows that the use of certain drugs may affect the function of the gastrointestinal tract, and may lead to a loss of bodily electrolytes and fluid [191]. Henceforth, limiting drug prescriptions to essential medications taken for as short a period as possible, and conducting periodic re-evaluations of the chosen treatment, are essential in order to minimize adverse drug–nutrient interactions.

## 5. Conclusions

In general, urinary tract infections are prevalent in society, and constitute a burden for the public health system. This review aimed to analyze the mechanisms related to the use of probiotics and bioactive substances from plant-based foods in the prevention of infections. Probiotics in their isolated form, present in fermented foods, as well as bioactive compounds present in vegetables and fruits, can possibly have a beneficial effect on health by indirectly preventing urinary tract infections.

However, despite these health effects, there is little evidence for a direct link between the consumption of probiotics and the prevention of UTIs, and further studies with better controls, statistical tests, and designs are required.

## Figures and Tables

**Table 1:** Biotherapeutics that display antimicrobial activity against uropathogens.

Biotherapeutic Name	Type	Mode of Action	Clinical Evidence	References
*E. coli* 83972	Asymptomatic Bacteriuria	Bacterial interference. Competition for nutrients. Biofilm interference. Bacteriocin production.	Phase 2—significant reduction in rUTI * (N = 20). No active phase 3. Approved for use in LUTD * by EAU *.	[23,24]
Lactin-V (*Lactobacillus crispatus*)	Probiotic	Lactic acid and hydrogen peroxide production. Blocks adherence to uroepithelial cells.	Phase 2—rUTI incidence rate reduced to 15% (N = 50). No active phase 3.	[25]
*L. rhamnosus* GG	Probiotic	Lactic acid and hydrogen peroxide production. Downregulates NF-κB, P, and Type 1 fimbriae. Biofilm interference.	Phase 2—one or two doses safe in NLUTD * and SCI * patients (N = 80). No active phase 3.	[26]
Mutaflor *(E. coli* Nissle 1917)	Probiotic	Microcin production. Competition for iron using iron uptake systems.	In vitro—no significant UPEC * reduction. Phase 4 in active development for children with UTIs.	[5,27]
Colicin E2	Bacteriocin	Endonucleolytic degradation of DNA.	Preliminary catheter trials—complete inhibition of susceptible *E. coli.*	[28]

* rUTI: recurrent urinary tract infection, LUTD: lower urinary tract dysfunction, EAU: European Association of Urology, NLUTD: neurogenic lower urinary tract dysfunction, SCI: spinal cord injury and UPEC: Uropathogenic *Escherichia coli*.

**Table 2:** Microorganisms, such as bacteria and some yeasts, that are often used as probiotics in foods.

Genera	Species	Benefits	Limitations
*Lactobacillus*	*acidophilus, casei, crispatus, delbrueckii subsp. bulgaricusa, fermentum, gasseri, johnsonii, paracasei, plantarum, reuteri, rhamnosus, helveticus, lactis, sporogenes*	Commonly used in fermented foods, including kimchi, sauerkraut, koumiss, yogurt, kurut, cheese, kefir, and kombucha.	There might be issues with interaction with medications that depress the immune system.
*Escherichia, Saccharomyces,* *Kluyveromyces, Streptococcus,* *Enterococcus* ^b^, *Propionibacterium,* *Pediococcus, Leuconostoc,* *Bacillus, Clostridium*	*Escherichia coli Nissle, Saccharomyces boulardii, S. cerevisiae, Kluyveromyces lactis, Streptococcus thermophilus* ^a^, *S. cremoris, S. diacetylactis, S. intermedius, S. salivarius, Enterococcus franciumb, Propionibacterium freudenreichii, P. freudenreichii subsp. shermanii, P. jensenii, L. lactis, Pediococcus, Leuconostoc lactis subsp. cremoris, L. lactis subsp. lactis, Bacillus cereus, Clostridium butyricum*	These bacteria and yeasts are commonly used in the dairy and brewing industries. They can improve the immune system in humans.	There is a need for more studies on patients with chronic diseases such as cancer.
*Bifidobacterium*	*bifidum, breve, infantis, longum, lactis, animalis, adolescentis, essensis, laterosporus*	Suitable as hosts for cellular engineering, to facilitate the increased bioproduction of value-added chemicals, while consuming fewer resources [30].	There is a need to better understand the impact and safety of their use to treat diseases.

^a^ poor survival during gastrointestinal transit, and ^b^ potential pathogenicity and vancomycin resistance, which were adapted from [31,32].

**Table 3:** Studies that correlate the consumption of cranberry, garlic, juniper, bearberry, and nettle with UTIs.

Plant Species	Common Name	Phytocompounds	Mode of Action	References
*Vaccinium macrocarpon*	Cranberry	Proanthocyanidins (PACs), malic acid, quinic acid, shikmic acid, hippuric acid, D-mannose, fructose	Decreases urinary pH; prevents bacterial adhesion to urinary tract; blocks bacterial receptors	[64,65,67,76,77,97,99]
*Allium sativum*	Garlic	Allicin, alliin, ajoene	Antibacterial; antifungal; prevents microorganisms from using some functions, like RNA and lipid biosynthesis; inhibits growth of *E. coli* and *Candida albicans*	[109,110,111,112,128,130,169]
*Juniperus communis*	Juniper	Essential oils (terpinen4-ol)	Diuretic properties; antimicrobial effects against UTI pathogens (*Klebsiella*, *Proteus*, and *Enterococcus*)	[139,152,153,154,155]
*Arctostaphylos uva-ursi*	Bearberry	Arbutin, ferulic acid, catechin, gallic acid, ellagic acid, caffeic acid	Diuretic action; inhibition of bacteria (*E. coli*, *S*. *aureus*, *Proteus vulgaris*, *Candida albicans*) through hydroquinone and alkalization of urine; prevention of bacterial attachment in urinary system	[79,136,137,138,139,140,141,143,144,147,149,150]
*Urtica dioica* L.	Nettle	Flavonoids, phenolic compounds, tannins, essential oils	Antimicrobial effect against Gram-positive and Gram-negative bacteria; diuretic properties due to compounds like caffeine, malic acid, and chlorogenic acid.	[157,158,159,160,161,162,163,164,166,167,168,170]
